# Competition for nitrogen between *Fagus sylvatica* and *Acer pseudoplatanus* seedlings depends on soil nitrogen availability

**DOI:** 10.3389/fpls.2015.00302

**Published:** 2015-04-30

**Authors:** Xiuyuan Li, Heinz Rennenberg, Judy Simon

**Affiliations:** ^1^Institute of Forest Sciences, Chair of Tree Physiology, University of FreiburgFreiburg, Germany; ^2^Plant Physiology and Biochemistry, Department of Biology, University of KonstanzKonstanz, Germany

**Keywords:** growth strategies, inorganic N uptake, inter-specific competition, intra-specific competition, N pools, organic N uptake, specific amino acids

## Abstract

Competition for nitrogen (N), particularly in resource-limited habitats, might be avoided by different N acquisition strategies of plants. In our study, we investigated whether slow-growing European beech and fast-growing sycamore maple seedlings avoid competition for growth-limiting N by different N uptake patterns and the potential alteration by soil N availability in a microcosm experiment. We quantified growth and biomass indices, ^15^N uptake capacity and N pools in the fine roots. Overall, growth indices, N acquisition and N pools in the fine roots were influenced by species-specific competition depending on soil N availability. With inter-specific competition, growth of sycamore maple reduced regardless of soil N supply, whereas beech only showed reduced growth when N was limited. Both species responded to inter-specific competition by alteration of N pools in the fine roots; however, sycamore maple showed a stronger response compared to beech for almost all N pools in roots, except for structural N at low soil N availability. Beech generally preferred organic N acquisition while sycamore maple took up more inorganic N. Furthermore, with inter-specific competition, beech had an enhanced organic N uptake capacity, while in sycamore maple inorganic N uptake capacity was impaired by the presence of beech. Although sycamore maple could tolerate the suboptimal conditions at the cost of reduced growth, our study indicates its reduced competitive ability for N compared to beech.

## Introduction

Plant species have evolved different strategies to maximize plant survival and reproduction by various combinations of physiological and morphological traits, depending on the environmental conditions (Reich et al., 1997, 2003; Craine et al., 2009). Many studies have investigated these different combinations of plant functional traits over the past decades with the focus on leaf traits and/or seed production (Reich et al., 2003), but knowledge on root traits is still scarce (Craine et al., 2009). Fast-growing species tend to have a higher photosynthetic capacity because of their higher light-capture area deployment per unit mass [high specific leaf area (SLA)] and faster turnover of plant parts, thus allowing flexibility in the plant’s response to the spatial heterogeneity of the environment (Reich et al., 1997; Westoby et al., 2002). This, in turn, ensures short-term advantages over slow-growing plants (Grime et al., 1994; Westoby et al., 2002), particularly in resource-limited environments in which competition is high. Thus, competition for resources, especially the growth-limiting macronutrient nitrogen, constitutes a major challenge for plants, including not only competition with soil microorganisms (Dannenmann et al., 2009; Rennenberg et al., 2009), but also other vegetation components, such as herbaceous and/or woody species (Fotelli et al., 2002, 2005; Simon et al., 2010b, 2011, 2014).

In Central Europe, European beech (*Fagus sylvatica*) represents the dominant tree species of the potential natural vegetation in moist to moderately dry areas of the sub-mountainous altitude range (Diekmann, 1996). Beech is favored by forest practitioners and governments because nowadays forest management practices have changed from supporting conifer monocultures to the preference of mixed species stands thereby promoting the natural regeneration of deciduous tree species (Fotelli et al., 2001, 2004; Petritan et al., 2009). The survival and growth of beech regeneration depends mainly on the ability to co-exist with highly competitive species (Tognetti et al., 1998; Fotelli et al., 2001, 2002, 2004, 2005). For example, Fotelli et al. (2002) showed that inorganic N uptake capacity of slow-growing beech seedlings was significantly reduced when grown together with the fast-growing pioneer shrub *Rubus fruticosus* and decreased even further with drought stress (Fotelli et al., 2004). Furthermore, other studies investigated the competition for nitrogen between beech and other potential competitors, such as soil microorganisms (Dannenmann et al., 2009), other tree species (Simon et al., 2010b, 2014), or even different developmental stages within a species (Simon et al., 2011). Beech seedlings and adult beech trees, for example, avoided competition for N by seasonal timing of N acquisition (Simon et al., 2011). Furthermore, in short-term studies investigating the competition for N between beech and sycamore maple seedlings (Simon et al., 2010b, 2014), we found evidence for different N uptake strategies that might depend on the growth strategies of the species.

Sycamore maple (*Acer pseudoplatanus*) – a relatively fast-growing species compared to slow-growing beech – might require large amounts of N by root uptake to meet its resource requirements for growth and development (Poorter et al., 2012). Because sycamore maple shares the spectrum where beech is dominant on calcareous substrate (Ellenberg, 1996), the two species might have evolved different strategies to successfully compete for nitrogen (Simon et al., 2010b) or avoid competition (Simon et al., 2011, 2014). Simon et al. (2010b) found that short-term competition between seedlings of both species lead to a reduced inorganic and organic N uptake capacity by slow-growing beech with limiting soil N, whereas inorganic N uptake capacity by fast-growing sycamore maple increased significantly. Under reduced light conditions, N acquisition by sycamore maple seedlings was negatively affected in the presence of beech indicating that beech is optimally attuned to shade conditions and outcompetes sycamore maple at least in short-term competition with reduced light availability (Simon et al., 2014). However, in these studies the focus was on short-term consequences (i.e., competing for 4 days), but not the implications of competition between two woody species when grown together for several months, a time during which competition might result in more distinct strategies of N uptake.

Therefore, this study aimed to elucidate (1) whether European beech and sycamore maple avoid competition for growth limiting N by different N uptake strategies, and (2) whether these strategies are altered by soil N availability. For this purpose, seedlings of European beech and sycamore maple were grown under controlled conditions in a microcosm experiment in which growth and biomass indices, N uptake capacity by the fine roots and N pools (i.e., total N, structural N, soluble protein-N, total amino acid-N, as well as specific amino acid-N) in the fine roots were analyzed. To ensure that differences in N uptake capacity were due to the other species, we set up the microorganisms also as intra-specific controls, i.e., with several individuals of the same species. Levels of specific amino acid-N in the roots were quantified to investigate overall patterns of potential differences in specific amino acid-N in the roots with competition and N supply.

## Materials and Methods

### Plant Material and Growth Conditions

Mycorrhizal seedlings of *F. sylvatica* L. (provenance Swabian Alb) and *A. pseudoplatanus* L. (provenance “Sueddeutsches Huegel-und Bergland montane Stufe”) of similar height (0.25–0.5 m) and structural characteristics were purchased from a commercial tree nursery (Schlegel & Co. Gartenprodukte GmbH, Riedlingen, Germany). One-year-old seedlings were chosen for this study, because the early developmental stage of seedlings is crucial for seedling establishment, in particular under competition for limited resources with other species (Madsen and Larsen, 1997; Zerbe, 2002). Microcosms (355 mm × 255 mm × 315 mm) were filled with a homogenous mixture of 0.7–1.2 mm silica sand (1 part), 0.1–0.5 mm silica sand (18 parts), perlite (19 parts), and torf (2 parts; Floragard Vertriebs GmbH für Gartenbau, Oldenburg, Germany) to keep plant available N from the soil substrate at a minimum. Seedlings were planted into the microcosms in November 2010 (see Experimental Design), over-wintered outside, and were transferred back to the greenhouse at the end of March 2011. Until the start of the experiment in mid-April, all microcosms were sufficiently irrigated every second day with an artificial low N solution (see below). Plants were grown under 16/8 h day/night conditions until the final harvest. Seedlings received natural daylight plus an artificial daylight supplied by mercury lamps (SON-T AGRO 400, Philips GmbH, Eindhoven, The Netherlands) with an average illumination intensity at canopy level of 412 ± 32 μmol m^-2^ s^-1^ (mean ± SD) during the day representing a tree-fall gap light environment (Tognetti et al., 1998). The average air temperatures were 21.1 ± 6.7 and 17.7 ± 3.8°C (day/night, mean ± SD). The average relative humidity was 41.5 ± 14.5% and 47.7 ± 7.1% (day/night, mean ± SD).

### Experimental Design

The experiment had a 3 × 2 factorial design with three levels of competitive interference (i.e., beech grown in intra-specific competition, sycamore maple grown in intra-specific competition, and beech and sycamore maple grown in inter-specific competition) and two levels of nitrogen supply (i.e., low or high) resulting in six treatment combinations. The level of competition was defined as: (1) European beech only (eight beech seedlings, BB), (2) sycamore maple only (eight sycamore maple seedlings, MM), and (3) beech and sycamore maple growing in competition (four beech plus 4 sycamore maple seedlings, BM). For each treatment, ten replicate microcosms were used containing eight seedlings planted with a tree to tree distance of c. 85 mm. This distance is well within the distance for neighboring plants to compete for resources (Gaudet and Keddy, 1988; Nernberg and Dale, 1997; Imo and Timmer, 1999). Two rows of three seedlings were arranged along each side and two seedlings were planted in the middle row of each microcosm. In microcosms containing both species, seedlings were spaced alternating by species to ensure that each individual was surrounded by individuals of the other species. Overall, the single species microcosms were used to study the effect of intra-specific competition and served as a control for the inter-specific competition. From the mixed species microcosms, both species were harvested and analyzed for inter-specific competition. This is shown in the results as BB-B (intra-specifically-grown beech), BM-B (beech grown in competition with sycamore maple), MM-M (intra-specifically grown sycamore maple), and BM-M (sycamore maple grown in competition with beech). BM-B and BM-M originate essentially from the same planting design, but for comparison purposes, a distinction is made whether the impact of sycamore maple on beech or beech on sycamore maple is being considered. After leaf development (mid-April), microcosms were separated into high/low N supply treatments and irrigated with 1 L of either low N (with a total of 151 μM N) or high N (with a total of 550 μM N) artificial nutrient solution every second day until the end of the experiment. The artificial low N solution was based on the soil solution at a low soil N field site in the Swabian Alb (Dannenmann et al., 2009) containing 100 μM KNO_3_, 90 μM CaCl_2_^∗^2H_2_O, 70 μM MgCl_2_^∗^6H_2_O, 50 μM KCl, 24 μM MnCl_2_^∗^4H_2_O, 20 μM NaCl, 10 μM AlCl_3_, 7 μM FeSO_4_^∗^7H_2_O, 6 μM K_2_HPO_4_, 1 μM NH_4_Cl, as well as the amino acids glutamine and arginine (25 μM each) at pH 6.5. The artificial high N solution was based on the soil solution of a high soil N field site in the Bavarian alpine upland containing 20 μM Al_2_(SO_4_)_3_, 75 μM CaCl_2_⋅2H_2_O, 4 μM FeCl_3_⋅6H_2_O, 14 μM KCl, 10 μM MnCl_2_⋅4H_2_O, 40 μM MgCl_2_⋅6H_2_O, 4.5 μM Na_2_HPO_4_, 20 μM NaCl, 50 μM NH_4_Cl, 300 μM KNO_3_, 100 μM glutamine, and 100 μM arginine at pH 4.7 (Stoelken et al., 2010). Glutamine and arginine were chosen as the most abundant amino acids in beech roots (Gessler et al., 1998).

### Harvest and Sample Preparation

Before the start of the experiment in mid-April, an initial harvest was conducted sampling three microcosms of each competition regime to determine the initial biomass and leaf area of seedlings required for calculation of relative growth rates (RGRs; see below). Ten weeks later, the final harvest was performed subsequent to ^15^N uptake experiments. At the initial and final harvest, seedlings were separated into fine roots, coarse roots, stems, and leaves which were oven-dried over 48 h at 65°C. Fresh and dry weights were determined. Leaf area was measured using an area meter (ΔT area meter, Delta-T devices, London, UK). In addition, fine root samples were shock-frozen in liquid nitrogen (after determining the fresh weight) and transferred to -80°C for storage until further processing. Prior to N metabolite quantification, frozen tissue was finely ground in liquid nitrogen.

### Growth and Biomass Allocation Indices

Average RGR was calculated for each seedling as RGR = (lnW_2_-lnW_1_)/(t_2_-t_1_), where W_1_ is the total plant biomass (g dw) per individual at the initial harvest of the experiment at t_1_ (day of the initial harvest), W_2_ is the total plant biomass (g dw) per individual at t_2_ (day of the final harvest; Grubb et al., 1996; Simon et al., 2010a). W_1_ was calculated from the average biomass of seedlings of each species (*n* = 24 grown in intra-specific competition, *n* = 16 grown in inter-specific competition). For other growth indices, data from t_2_ was used, including SLA, leaf mass ratio (LMR), and leaf area ratio (LAR, total leaf area as a proportion of total plant biomass). Net assimilation rate (NAR) was calculated by dividing RGR by LAR. Leaf nitrogen productivity (LNP, an index of plant growth relative to leaf N) was determined according to: LNP = RGR/(N_a_^∗^ LAR), where N_a_ is the foliar nitrogen concentration per unit leaf area (Simon et al., 2010b). Furthermore, root:shoot ratio (R: S_m_) was determined on a mass basis.

### ^15^N Uptake Experiments

For the ^15^N uptake experiments, the two center seedlings and in addition the middle one of each site from the inter-specific competition microcosms were chosen. The ^15^N enrichment technique as described by Gessler et al. (1998) and Simon et al. (2010b) was applied to determine N uptake capacity. Both long sides of the microcosm were cut open for easy access to the fine roots. Fine roots still attached to the plants were carefully dug out and rinsed with distilled water to remove adhering substrate particles. To quantify N uptake rates, roots were incubated for 2 h (between 10:00 am to 14:00 pm to avoid diurnal variation in N uptake (Gessler et al., 2002) in 4 mL of either low or high N solution (see above) with one of four N compounds labeled either as ^15^NO_3_^-^, ^15^NH_4_^+^, or ^15^N/^13^C double-labeled glutamine or arginine, or control solutions without ^15^N label (to account for the natural abundance of ^15^N in the fine roots). After 2 h incubation, the submersed root tips and moistened upper parts (∼8–10 mm) were cut off, washed twice with 0.5 μM CaCl_2,_ dried out with cellulose paper and oven-dried for 48 h at 65°C. Fresh and dry weight was determined.

### Quantification of ^15^N, ^13^C, and Total N Amounts in Fine Roots and Leaves

For the determination of ^15^N and ^13^C abundance and total N in fine roots and leaves, the dried tissue (48 h, 60°C) was ground into a fine homogeneous powder using a ball mill. Aliquots of 1.2–2 mg were transferred into tin capsules (IVA Analysentechnik, Meerbusch, Germany) and analyzed using an elemental analyzer (NA2500, CE Instruments, Milan, Italy), coupled via a Conflo II interface to an isotope ratio mass spectrometer (Delta Plus, Thermo Finnigan MAT GmbH, Bremen, Germany). Working standards (glutamic acid), calibrated against the primary standards USGS 40 (Glutamic acid, δ^13^C_PDB_ = -26.39) and USGS 41 (Glutamic acid, δ^13^C_PDB_ = 37.63) for δ^13^C and USGS 25 (ammonium sulfate, δ^15^N_Air_ = -30.4) and USGS 41 (δ^15^N_Air_ = 47.600) for δ^15^N, were analyzed after every 12th sample to detect a potential instrument drift over time.

N uptake capacity (nmol g^-1^ fw h^-1^) was calculated based on the incorporation of ^15^N into fine roots and the respective plant biomass according to the equation by Geßler et al. (1998): N uptake capacity = ((^15^N_l_-^15^N_c_)^∗^N_tot_^∗^dw^∗^10^5^)/(MW^∗^fw^∗^t), where ^15^N_l_ and ^15^N_c_ are the atom% of ^15^N in labeled (N_l_) and control plants (N_c_, natural abundance), respectively, N_tot_ is the total N percentage, and MW is the molecular weight (^15^N g mol^-1^), t represents the incubation time (120 min). Based on ^13^C incorporation into the root fresh weight, the uptake rates of amino acids were generally lower compared to those based on the ^15^N incorporation indicating either the degradation of amino acids in the incubation solution or on the root surface, or respiration of amino acid-derived carbon inside the roots (Simon et al., 2011).

### Quantification of Total Soluble Protein in Fine Roots

Total soluble proteins in fine roots were quantified according to the method by Dannenmann et al. (2009). Frozen fine ground roots (c. 50 mg) were extracted in 1 mL extraction buffer (50 mM Tris-Cl (pH 8.0), 1 mM EDTA, 15% glycerol (v:v), 1 mM phenylmenthylsulfonyl fluoride, 5 mM dithiothreitol, 0.1% Triton X-100). After 30 min incubation on the shaker at 4°C followed by centrifugation at 14,000 *g* for 10 min at 4°C, 500 μL trichloroacetic acid (10%) were added to 500 μL aliquots of the supernatant, and then incubated for 10 min at room temperature. After centrifugation at 14,000 *g* for 10 min, the supernatant was carefully discarded and the protein pellets were dissolved in 0.5 mL 1 M KOH. Bradford reagent (1 mL; Amresco Inc., Solon, OH, USA) was added to 50 μL aliquots of the extracts for quantification of total soluble protein. After 10 min of incubation at room temperature in the dark, the optical density was measured in a UV-DU650 spectrophotometer (Beckman Coulter Inc., Fullerton, CA, USA) at 595 nm. Bovine serum albumin (BSA, sigma A-6918) was used as a standard.

### Quantification of Total and Specific Amino Acids and Ammonium in Fine Roots

Amino acids and ammonium were extracted according to the method of Winter et al. (1992). Aliquots of c. 50 mg frozen, homogenized root tissue were extracted in 0.2 mL buffer (20 mM Hepes, 50 mM EGTA, 10 mM NaF, pH 7.0) and 1 mL methanol: chloroform (3.5:1.5, v:v). After shaking for 30 min at 4°C, 600 μL distilled H_2_O were added to the samples, mixed, and centrifuged for 5 min at 4°C. This extraction step was repeated once. The quantification of total amino acid was determined from the combined supernatants according to the method by Liu et al. (2005). For quantification of total amino acids, aliquots (100 μL) of the supernatant and 100 μL of ninhydrin reagent a 50:50 (v:v) mixture of solution A (containing 4.2 g citric acid⋅H_2_O, 0.16 g SnCl_2_⋅2H_2_O, and 40 mL 1 M NaOH, made up to 100 mL with distilled water at pH 5) and solution B containing 4 g ninhydrin in 100 mL ethylene glycol monomethyl ether) were boiled at 95°C for 30 min. Isopropanol (1.25 mL, 50%) was added to the mixture followed by 15 min incubation. The optical density was measured using a DV-UV650 spectrophotometer (Beckman Coulter Inc., Fullerton, CA, USA) at 570 nm. Glutamine was used as a standard. For quantification of specific amino acids and ammonium, the extracts were shock-frozen in liquid N_2,_ and freeze-dried for 96 h. The composition and concentration of amino compounds and ammonium was determined in 50 μL extracts analyzed with a Water Acquity UPLC-System (Waters Corp., Milford, MA, USA) using a modified standard protocol (using an AccQ-Tag^TM^ Ultra column 2.1 mm × 100 mm, 1.7 μm, 0.7 mL/min flow, column temperature 61°C) as previously described (Luo et al., 2009). Amino acid Standard H (#NCI0180, Pierce Biotechnology, Inc., Rockford, IL, USA) was used as an analytical standard plus additional specific amino acids and ammonium added (with 2.5 μmol in 0.1 N HCl each) according to the composition of the analyzed sample.

### Quantification of Nitrate in Fine Roots

Nitrate was quantified according to the method described by (Dannenmann et al., 2009). Approximately 100 mg washed polyvinylpyrrolidone (PVPP, Sigma-Aldrich Inc., Steinheim, Germany) were soaked in 1 mL distilled H_2_O overnight. About 50 mg root material was added to the solution and shaken for 1 h in the dark. Samples were boiled at 95°C for 10 min, followed by 10 min centrifugation at 4°C. Aliquots of 150 μL supernatant were measured in an auto-sampler (AS3500, Thermo Separation Products, Piscataway, NJ, USA) connected with an ion chromatography system (DX120, Dionex, Idstein, Germany). The ion chromatography system was equipped with a guard column (RFIC^TM^ IonPac AS9-SC, 4 mm × 250 mm, Dionex, Idstein, Germany), an analytical column (IonPac AS9-SC, 4 mm × 250 mm, Dionex, Idstein, Germany) and a self-regenerating suppressor (ASRS-ULTRA II, 4 mm, Dionex, Idstein, Germany). An eluent solution of 2.0 mM sodium carbonate and 0.75 mM sodium bicarbonate was used for the separation of different anions. An anion mixture of NO_3_^-^, PO_4_^3-^, SO_3_^2-^, and SO_4_^2-^ was used as a standard.

### Statistical Analyses

For all measured parameters, normality tests and Levene’s test for homogeneity of variances were performed using SPSS 16.0 (SPSS Inc., Chicago, IL, USA). To detect differences among treatments, two-way analyses of variance (ANOVAs) were conducted using SigmaPlot 12.5 (Systat Software Inc., San Jose, CA, USA). Two factors were defined as (1) high/low N supply and (2) competition regimes. Holm-Sidak *post hoc* test was performed subsequently to compare differences within each factor by SigmaPlot (Systat Software Inc., San Jose, CA, USA). Datasets of specific amino acids were subjected to principal component analysis (PCA) and partial least squares discriminant analysis (PLS-DA) using MetaboAnalyst 2.0 (Xia et al., 2012).

## Results

### Combined Influence of Soil N Availability and Competition Regime on Growth Indices, N Acquisition and N Pools

To study the combined influence of soil N availability and competition regime on growth indices, N acquisition and N pools in the fine roots of beech and sycamore maple seedlings, two-way ANOVAs were performed. Only R:S_m_, LAR, foliar N_a_, ammonium, and Arg-N uptake capacity (*P* ≤ 0.030), as well as nitrate-N and soluble protein-N pools in the fine roots (*P* ≤ 0.023) were significantly affected by the combination of both treatments (**Table 1**).

**Table 1:** Two-way ANOVA analyses of growth indices, N uptake capacity and N pools in the fine roots of beech and sycamore maple seedlings.

	N supply ^∗^ competition	N supply	Competition
**Growth indices**
R:S_m_	**0.018**	0.292	0.241
RGR (mg g^-1^ d^-1^)	0.600	0.101	**<0.001**
NAR (g m^2^ d^-1^)	0.211	0.658	**<0.001**
LNP (g g(N_m_)^-1^ d^-1^)	0.212	0.058	**<0.001**
LMR (g g^-1^)	0.905	**0.016**	**<0.001**
LAR (cm^2^ g^-1^)	**0.030**	0.186	**0.004**
SLA (m^2^ g^-1^)	0.412	0.419	**<0.001**
N_a_ (g m^-2^)	**<0.001**	**<0.001**	**<0.001**
**N uptake capacity**
Ammonium	**<0.001**	**<0.001**	**<0.001**
Nitrate	0.496	**<0.001**	**<0.001**
Arginine-N	**<0.001**	**<0.001**	**<0.001**
Glutamine-N	0.172	**<0.001**	**<0.001**
**N pools**
Total N	0.123	0.283	**<0.001**
Structural N	0.099	0.120	0.153
Total soluble protein-N	**0.023**	**< 0.001**	**0.031**
Total amino acid-N	0.226	**<0.001**	**0.001**
Nitrate-N	**<0.001**	**<0.001**	**<0.001**
Ammonium-N	0.494	**0.004**	**<0.001**

The effects of all parameters were separated by two factors (1) N supply and (2) competition regimes. R:S_*m*_, root/shoot mass ratio; RGR, relative growth rate; NAR, net assimilation rate; LNP, leaf nitrogen productivity; LMR, leaf mass ratio; LAR, leaf area ratio; SLA, specific leaf area; N_*a*_, nitrogen concentration per unit leaf area. Bold values indicate the significance level of 0.050.

### Consequences of Soil N Availability on N Acquisition and N Pools in Both Species

**FIGURE 1:**
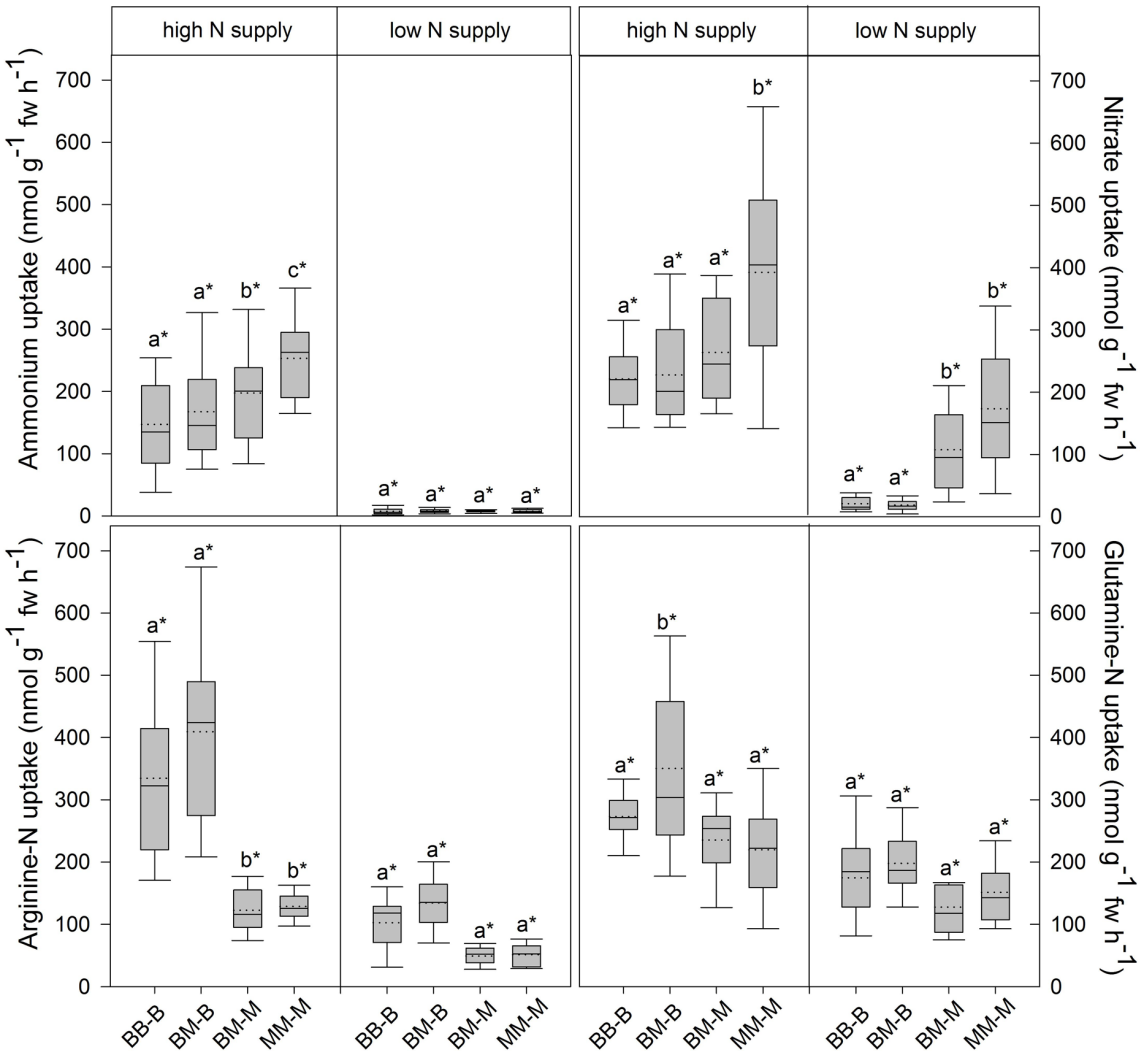
**Inorganic and organic N uptake capacity (nmol g^-1^ fw h^-1^) in beech and sycamore maple seedlings with high or low soil N supply.** BB-B: beech grown in intra-specific competition, BM-B: beech grown in competition with sycamore maple, BM-M: sycamore maple grown in competition with beech, MM-M: sycamore maple grown in intra-specific competition. Box plots show means (dotted lines) and medians (straight lines; *n* = 14 for each treatment). Different small letters indicate significant differences between the four competition regimes (i.e., BB-B, BM-B, BM-M, MM-M) within one soil N supply (*P* ≤ 0.050). Asterisk indicates significant difference between high and low soil N supply within one competition regime (*P* ≤ 0.050).

**FIGURE 2:**
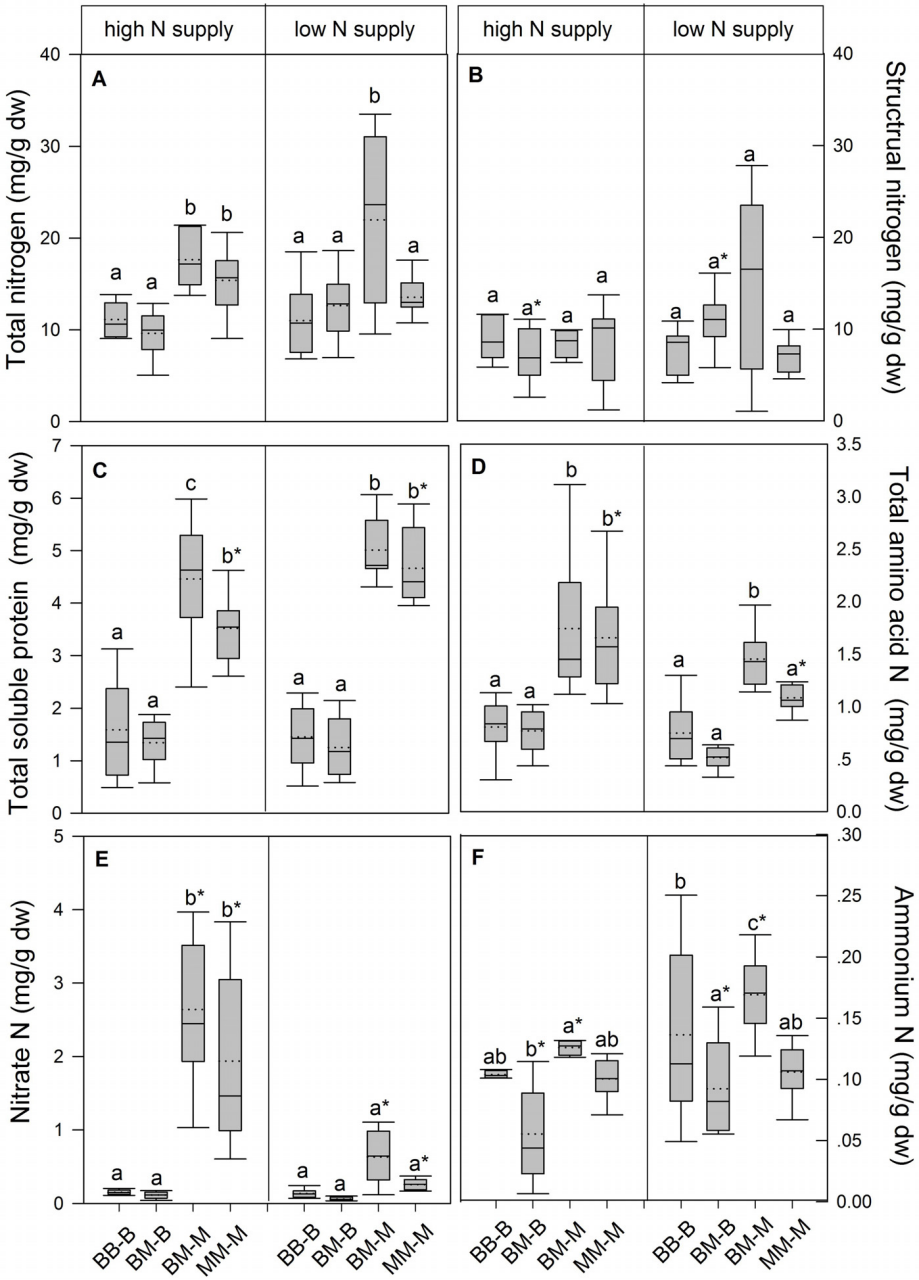
**Nitrogen pools (mg g^-1^ dw) in the fine roots of beech and sycamore maple seedlings with high or low soil N supply. (A)** Total N, **(B)** structural N, **(C)** total soluble protein-N, **(D)** total amino acid-N, **(E)** nitrate-N, **(F)** ammonium-N. BB-B: beech grown in intra-specific competition, BM-B: beech grown in competition with sycamore maple, BM-M: sycamore maple grown in competition with beech, MM-M: sycamore maple grown in intra-specific competition. Box plots show means (dotted lines) and medians (straight lines; *n* = 14 for each treatment). Different small letters indicate significant differences between the competition regimes (i.e., BB-B, BM-B, BM-M, MM-M) within one soil N supply (*P* ≤ 0.050). Asterisks indicate significant differences between high and low soil N supply within one competition regime (*P* ≤ 0.050).

In European beech seedlings, decreasing soil N availability (i.e., high vs. low N supply) resulted in significant changes in N uptake capacity and N pools in the fine roots in both competition regimes (**Table 1**). No significant differences with N supply were found for any of the growth indices except for an increase in foliar N_a_ in beech regardless of competition regime with decreasing soil N supply (*P<*0.001; **Tables 1** and **2**). Furthermore, decreasing soil N availability led to declining N uptake capacities for all four tested N sources regardless of competition regime (*P* < 0.001; **Table 1**; **Figure 1**). With regard to N pools in the fine roots of beech, only beech seedlings grown in inter-specific competition had higher levels of ammonium-N with low compared to high N supply (*P*= 0.037; **Figure 2**).

**Table 2:** Biomass and leaf parameters of beech and sycamore seedlings with high/low soil N supply (mean ± SD).

	High N supply				Low N supply			
	BB-B	BM-B	BM-M	MM-M	BB-B	BM-B	BM-M	MM-M
R:S_m_	0.832 ± 0.109^a^	0.891 ± 0.141^a^	0.780 ± 0.195^a^	0.874 ± 0.204^a^	0.906 ± 0.171^a^	0.873 ± 0.198^a^	0.865 ± 0.173^a^	0.813 ± 0.196^a^
RGR (mg g^-1^ d^-1^)	6.232 ± 5.179^b^	3.325 ± 6.559^ab^	1.841 ± 6.303^a^	12.685 ± 4.525^c^	4.587 ± 7.278^b^	0.866 ± 5.721^a^	1.912 ± 6.559^ab^	10.581 ± 5.724^c^
NAR (g m^2^ d^-1^)	0.178 ± 0.148^b^	0.095 ± 0.148^ab^	0.101 ± 0.26^a^	0.417 ± 0.153^c^	0.148 ± 0.235^b^	0.027 ± 0.175^a^	0.080 ± 0.269^ab^	0.395 ± 0.214^c^
LNP (g g(N_m_)^-1^ d^-1^)	1.667 ± 1.700^b^	0.847 ± 0.995^ab^	1.349 ± 2.751^a^	4.932 ± 1.189^c^	0.943 ± 0.985^b^	0.014 ± 0.041^a^	0.800 ± 1.936^ab^	4.574 ± 2.051^c^
LMR (g g^-1^)	0.170 ± 0.034^b^	0.180 ± 0.036^b^	0.106 ± 0.028^a^	0.105 ± 0.021^a^	0.162 ± 0.023^b^	0.168 ± 0.021^b^	0.102 ± 0.020^a^	0.097 ± 0.020^a^
LAR (cm^2^ g^-1^)	34.937 ± 5.569^a^	35.091 ± 23.746^a^	30.644 ± 9.375^a∗^	29.644 ± 6.925^a^	30.913 ± 5.380^a^	32.604 ± 6.806^a^	24.245 ± 6.106^a∗^	26.807 ± 9.093^a^
SLA (m^2^ g^-1^)	0.318 ± 0.027^a^	0.334 ± 0.036^a^	0.421 ± 0.127^b^	0.399 ± 0.083^b^	0.326 ± 0.032^a^	0.327 ± 0.035^a^	0.375 ± 0.095^ab^	0.404 ± 0.036^b^
N_a_ (g m^-2^)	1.190 ± 0.104^b∗^	1.201 ± 0.077^b∗^	0.809 ± 0.025^a∗^	1.057 ± 0.158^b^	1.594 ± 0.112^c∗^	1.408 ± 0.120^c∗^	1.341 ± 0.045^b∗^	1.000 ± 0.105^a^

R:S_*m*_, root/shoot mass ratio; RGR, relative growth rate; NAR, net assimilation rate; LNP, leaf nitrogen productivity; LMR, leaf mass ratio; LAR, leaf area ratio; SLA, specific leaf area; N_*a*_, nitrogen concentration per unit leaf area. BB-B: beech grown in intra-specific competition, BM-B: beech grown in inter-specific competition with sycamore maple, BM-M: sycamore maple grown in inter-specific competition with beech, MM-M: sycamore maple grown in intra-specific competition. Different small letters indicate significant differences between the competition regimes (i.e., BB-B, BM-B, BM-M, MM-M) within one soil N supply treatment (*P* ≤ 0.05). Asterisks indicate significant differences between high and low soil N supply within one competition regime (*P* ≤ 0.05).

Similar to beech, N uptake capacity in sycamore maple generally declined with decreasing soil N supply (*P* ≤ 0.012; **Figure 1**). Furthermore, with declining soil N availability, levels of nitrate-N (regardless of competition regime) and total amino acid-N (only with intra-specific competition) significantly decreased (*P* ≤ 0.001), whereas levels of soluble protein-N (with intra-specific competition) and ammonium-N (with inter-specific competition) increased (*P* ≤ 0.040; **Figure 2**) in sycamore maple. With regard to growth indices, LAR decreased (*P*= 0.023) and foliar N_a_ increased (*P* <0.001) significantly with decreasing soil N supply in sycamore maple only with inter-specific competition.

### Consequences of Competition Regime on Growth Indices, N Acquisition and N Pools in European Beech Seedlings

Beech seedlings responded to inter-specific competition with sycamore maple with regard to growth indices, N uptake capacity and N pools in the fine roots (**Table 2**; **Figures 1** and **2**). Beech showed no significant differences of biomass by the influence of sycamore maple under high N supply, whereas under low N supply RGR, NAR, and LNP decreased in presence of sycamore maple (*P* ≤ 0.032; **Table 2**). Generally, for beech, organic N (i.e., Gln and Arg) was the preferred N source regardless of soil N availability and competition regime (*P* ≤ 0.001; **Figure 1**). With high soil N availability, competition with sycamore maple only led to an increase in Gln-N and Arg-N uptake capacity (*P* ≤ 0.033) in beech, but exhibited no changes in ammonium and nitrate uptake capacity (**Figure 1**) compared to intra-specific competition. With regard to N pools in beech roots, ammonium-N concentration in beech roots decreased when grown with sycamore maple with low N supply (*P*= 0.044), while no other changes in N pools in beech were found at high or low soil N availability (**Figure 2**).

### Consequences of Competition Regime on Growth Indices, N Acquisition and N Pools in Sycamore Maple Seedlings

Similar to beech, sycamore maple seedlings responded with changes in N uptake capacity and N pools in the fine roots when grown in competition with beech, but also showed differences in growth and biomass indices. At high soil N availability, RGR, NAR, LNP, and foliar N_a_ decreased was significantly in the presence of beech (*P* ≤ 0.008; **Table 2**). Furthermore, inorganic N uptake capacity (i.e., nitrate, ammonium) were significantly lower in sycamore maple in the presence of beech (*P* ≤ 0.047; **Figure 1**) except for ammonium with low N supply, whereas no changes were found for organic N uptake. With regard to N pools in the fine roots, concentrations of soluble protein-N and nitrate-N increased in sycamore maple in the presence of beech at high soil N availability, while no other changes in N pools in beech were found (*P* ≤ 0.014; **Figures 2C,E**).

At low soil N supply, sycamore maple had reduced RGR, NAR, and LNP (*P* ≤ 0.001), whereas foliar N_a_ increased (*P* ≤ 0.001) when grown in competition with beech (**Table 2**). Similar to high N supply, nitrate-N uptake capacity in sycamore maple decreased in the presence of beech. Concentrations of total N, soluble protein, total amino acids and ammonium N increased significantly in the fine roots of sycamore maple grown in competition with beech at low soil N supply (*P* ≤ 0.004; **Figure 2**).

### Comparing European Beech and Sycamore Maple Seedlings

The responses to inter/intra-specific competition with regard to growth and biomass indices, as well as N uptake capacity and N metabolites in the fine roots between the two species, differed depending on species and soil N supply. At intra-specific competition, beech had lower RGR, NAR, LNP, and SLA but higher LMR than sycamore maple independent of soil N availability (*P* ≤ 0.016; **Table 2**). However, when beech and sycamore maple were grown in inter-specific competition, both species showed similar R:S_m_, RGR, NAR, LNP, and LAR regardless of soil N supply. Comparing beech and sycamore maple in inter-specific competition, with high soil N supply, beech seedlings had a lower SLA and higher N_a_ and LMR (*P* ≤ 0.002; **Table 2**), whereas at low soil N supply no significant differences were found for SLA between beech and sycamore maple in inter-specific competition.

Beech showed lower inorganic N uptake capacity than sycamore maple in intra-specific competition regardless of N supply, except for ammonium with low N supply (*P* <0.001; **Figure 1**). Beech and sycamore maple grown in inter-specific competition, showed similar inorganic N uptake, except for nitrate which was still higher in sycamore maple than in beech (*P*= 0.019; **Figure 1**). Regarding organic N uptake capacity, Arg-N uptake capacity was generally higher in beech compared to sycamore maple regardless of N availability and competitive regimes (*P* ≤ 0.004), except for beech and sycamore maple in intra-specific competition with low N supply. With high N supply, Gln-N uptake capacity increased in beech in presence of sycamore maple.

With regard to N metabolites in the fine roots, beech seedlings had lower concentrations of total N (except for that in intra-specific competition at low N supply), nitrate-N (only at high N supply), total soluble protein-N, total amino-acid-N and ammonium-N (only in inter-specific competition; *P* ≤ 0.001) than sycamore maple seedlings, regardless of soil N availability and competition regime (**Figure 2**).

**FIGURE 3:**
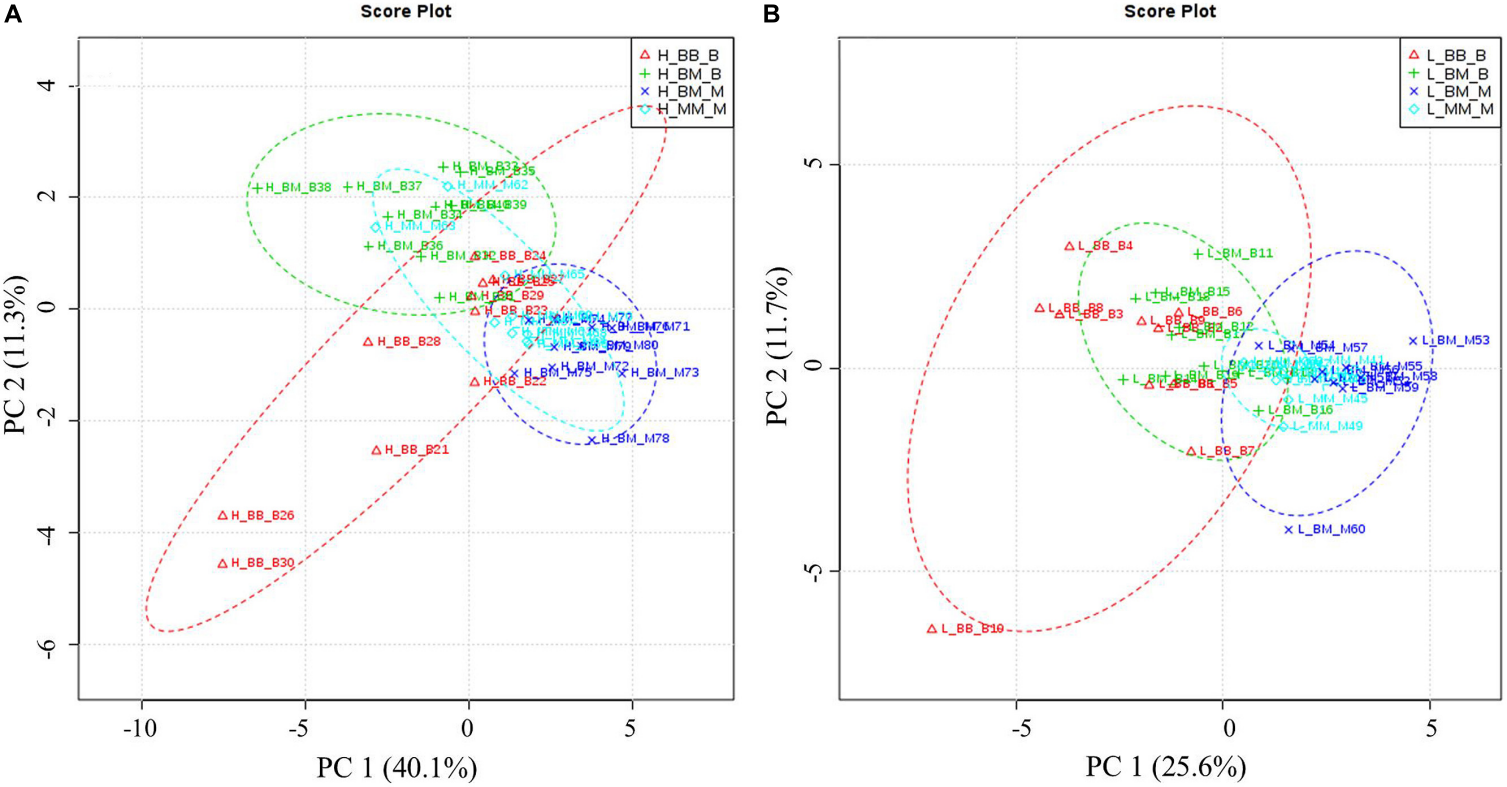
**Principal components analysis (PCA) based on specific amino acid concentration.** H: with high soil N supply **(A)**, L: with low soil N supply **(B)**. BB-B: beech grown in intra-specific competition, BM-B: beech grown in competition with sycamore maple, BM-M: sycamore maple grown in competition with beech, MM-M: sycamore maple grown in intra-specific competition. **(A)** PCA of specific amino acids levels under high soil N supply. According to the variance of the specific amino acids along PC1 and 2 (capture 51.4% of the total variances), the groups of beech and maple under inter-specific competition were separated distinctly. **(B)** PCA of specific amino acids levels under low soil N supply. Beech and sycamore maple under inter-specific competition are grouped with a little part overlapped (PC1 and 2 explain 37.3% of variance).

Principal component analysis showed that competition regime and soil N availability led to overall changes in amino acid-N composition in the fine roots of beech and sycamore maple (**Figure 3**). With high N availability, the influence of intra-specific competition showed overlapping areas in beech and sycamore maple, whereas the presence of the competing species led to species-specific amino acid composition. This species specificity became more pronounced when N supply was limited, and both species showed similar composition regardless of competition regime.

## Discussion

### Response of Beech Seedlings to Competition for N Depends on Soil N Availability

For beech seedlings, the consequences of competition for N with sycamore maple had no direct influence on growth indices with high N supply, but physiological changes were found regardless of soil N availability when comparing with intra-specifically grown beech seedlings. This indicates that the underlying mechanisms regulating growth patterns might shift depending on the competing species. Comparing the N acquisition strategies of beech seedlings grown in intra- vs. inter-specific competition, with high soil N availability, beech seedlings showed an increased Gln-N and Arg-N uptake capacity when competing with sycamore maple. This strategy is in contrast to previous results from a study investigating the consequences of short-term competition for N between beech and sycamore maple seedlings (Simon et al., 2010b). In this study, beech seedlings had significantly lower inorganic and organic N uptake capacity in the presence of maple which might have been due to the release of an inhibiting compound by sycamore maple (Simon et al., 2010b). A similar study using the same composition of N compounds applied to the roots and investigating the effect of light availability on the competition between beech and sycamore maple also under short-term conditions (Simon et al., 2014) could not show the inhibiting effect of sycamore maple on beech. Thus, it is still unclear, under what conditions inhibition of N uptake in beech seedlings might occur. The experimental set-up of the experiments conducted on short-term competition (Simon et al., 2010b, 2014) is not directly comparable with the present study. The results of our present study indicate that under close to natural conditions beech does not have a disadvantage, but might have rather adapted to the competition by increasing organic (i.e., Gln and Arg) N uptake. This preference of organic over inorganic N is consistent with other studies on beech roots (e.g., Dannenmann et al., 2009; Simon et al., 2010b, 2011, 2014). N pools in the fine roots of beech did not change in the presence of sycamore maple regardless of N supply, except for a decrease of ammonium-N concentration in beech in the presence of sycamore maple with limited N supply. However, we measured only N pools in fine roots, thus differences in levels of N metabolites might also be due to metabolite transport from above ground tissues (Herschbach et al., 2012). These changes in N acquisition and allocation to N pools in the fine roots indicate that different N use strategies of beech in the presence of sycamore maple depend on soil N supply. Furthermore, N acquisition of beech seedlings was adapted to the competition with sycamore maple regardless of N supply, because N uptake capacity of beech was not impaired.

### Response of Sycamore Maple Seedlings to Competition for N Varies with Competition Regime and Soil N Availability

For sycamore maple, competition led to a reduction in RGR due to lower LNP and NAR (both regardless of soil N availability) in sycamore maple seedling regardless of soil N supply, thus showing a visible response to competition with beech seedlings. The reduction in inorganic N acquisition by sycamore maple (regardless of N supply) indicates that sycamore maple might be outcompeted in competition by beech as was also indicated in the short-term competition with decreasing light availability (Simon et al., 2014). Apparently, N acquisition strategies of sycamore maple depend not solely on abiotic stressors but shift with abiotic–biotic stressor combination, e.g., decline in N uptake capacity with reduced light availability in short-term competition (Simon et al., 2014), but regardless of N availability in competition (present study). Sycamore maple tended to prefer inorganic N (i.e., ammonium and nitrate) at high soil N availability regardless of competition regime in contrast to results found for beech seedlings (Stoelken et al., 2010). However, limited N availability also led to a general decrease in N uptake capacity as found in beech (Stoelken et al., 2010). In contrast to beech seedlings, total N, soluble protein-N, total amino acid-N, and ammonium-N concentrations in the fine roots of sycamore maple increased at low soil N availability when seedlings were grown in competition with beech suggesting that sycamore maple responded stronger to the change in soil N supply compared to beech. The levels of soluble protein-N in the fine roots of sycamore maple increased when grown in competition with beech confirming the results from previous experiments that sycamore maple – when grown in competition with beech (Simon et al., 2010b, 2014) – synthesizes proteins, probably representing a specific adaptation of sycamore maple.

### Different Strategies of Competition – Beech vs. Sycamore Maple

Beech and sycamore maple seedlings showed different responses to soil N availability with regard to growth, N acquisition and composition of N pools in the fine roots, similar to different responses to changing light availability investigated in a previous study (Simon et al., 2014). Furthermore, the responses were also influenced by competition between the two species in the present study. When grown in intra-specific competition, beech and sycamore maple show different growth strategies at least at the seedling level. Beech is a relatively slow-growing, whereas sycamore maple is a relatively fast growing species (Ellenberg, 1996), represented in lower LNP, SLA, and NAR regardless of soil N availability. The species-specific differences in N acquisition, namely a higher organic N uptake capacity for beech seedlings than in sycamore maple, and a higher inorganic N uptake capacity in sycamore maple than in beech, confirm the theory that competition for N can be avoided (e.g., Simon et al., 2011; Hodge and Fitter, 2013). These contrasting results compared to previous studies investigating the competition for N between beech and sycamore maple grown in short-term competition (Simon et al., 2010b, 2014) indicate that N uptake strategies might shift over time. Whereas in short-term competition, plant species might actually compete for limited resources, in the longer run they acclimate to the conditions and develop an avoidance strategy. Species differed also in their N pools in the fine roots regardless of soil N availability and competition regime. Sycamore maple had generally higher levels of total N, total soluble protein-N, total amino acid-N and nitrate-N compared to beech. Furthermore, the different approaches to cope with low soil N availability (i.e., differences in N allocation to fine root N pools) suggest that beech is better adapted to N limitation compared to sycamore maple. In addition, the present results show that competition in sycamore maple led to an increase in soluble protein concentration when grown with beech. This is consistent with previous studies (Simon et al., 2010b, 2014). Further experiments are required to test whether this common strategy is a consequence of *de novo* synthesis of proteins involved in the interaction between the two competing species. Soil N availability and the presence of a competing species also resulted in shifts in amino acid-N composition in the fine roots in both species. With high N supply, competition led to species-specific amino acid-N composition, whereas with intra-specific competition similar patterns were found suggesting that amino acid compositions shift depending on the competition regime even when N is available in excess. With N limitation, however, these patterns became less pronounced, because both had similar amino acid composition regardless of the competition regime indicating that soil N availability plays a major role in the competition for N between species.

## Conclusion

Growth, N acquisition, and nutrition strategies of relatively slow-growing beech and relatively fast growing maple are adapted to N availability in the soil. The present results indicate that in beech-dominated forests on low N soil, the dominant tree species – beech – is optimally adapted to the environment. Intra- or inter-specific competition for N is avoided by different preferences for N sources in N acquisition. Furthermore, the understory provides the optimal environment to support beech seedlings rather than other woody plant species, such as sycamore maple which appears to be able to tolerate the conditions but at the cost of reduced growth and N acquisition capacity, therefore losing its competitive ability over beech. However, field studies are required to confirm these findings for the competition between beech and sycamore maple. Whether similar mechanisms of competition have been developed with other woody competitors, remains to be investigated.

## Author Contributions

JS and XL conceived the experiment. XL conducted the experiment and analyzed the data. XL and JS wrote the paper. HR contributed ideas and improved the manuscript.

## Conflict of Interest Statement

The authors declare that the research was conducted in the absence of any commercial or financial relationships that could be construed as a potential conflict of interest.
